# Weighing the waitlist: Weight changes and access to kidney transplantation among obese candidates

**DOI:** 10.1371/journal.pone.0242784

**Published:** 2020-11-30

**Authors:** Elaine Ku, Adrian M. Whelan, Charles E. McCulloch, Brian Lee, Claus U. Niemann, Garrett R. Roll, Barbara A. Grimes, Kirsten L. Johansen

**Affiliations:** 1 Division of Nephrology, Department of Medicine, University of California, San Francisco, California, United States of America; 2 Division of Pediatric Nephrology, Department of Pediatrics, University of California, San Francisco, California, United States of America; 3 Department of Epidemiology and Biostatistics, University of California, San Francisco, California, United States of America; 4 Department of Anesthesia and Perioperative Care, University of California, San Francisco, California, United States of America; 5 Division of Transplant Surgery, Department of Surgery, University of California, San Francisco, California, United States of America; 6 Division of Nephrology, Department of Medicine, Hennepin County Medical Center, Minneapolis, Minnesota, United States of America; 7 Department of Medicine, University of Minnesota, Minneapolis, Minnesota, United States of America; Imperial College Healthcare NHS Trust, UNITED KINGDOM

## Abstract

High body mass index is a known barrier to access to kidney transplantation in patients with end-stage kidney disease. The extent to which weight and weight changes affect access to transplantation among obese candidates differentially by race/ethnicity has received little attention. We included 10 221 obese patients waitlisted for kidney transplantation prior to end-stage kidney disease onset between 1995–2015. We used multinomial logistic regression models to examine the association between race/ethnicity and annualized change in body mass index (defined as stable [-2 to 2 kg/m^2^/year], loss [>2 kg/m^2^/year] or gain [>2 kg/m^2^/year]). We then used Fine-Gray models to examine the association between weight changes and access to living or deceased donor transplantation by race/ethnicity, accounting for the competing risk of death. Overall, 29% of the cohort lost weight and 7% gained weight; 46% received a transplant. Non-Hispanic blacks had a 24% (95% CI 1.12–1.38) higher odds of weight loss and 22% lower odds of weight gain (95% CI 0.64–0.95) compared with non-Hispanic whites. Hispanics did not differ from whites in their odds of weight loss or weight gain. Overall, weight gain was associated with lower access to transplantation (HR 0.88 [95% CI 0.79–0.99]) compared with maintenance of stable weight, but weight loss was not associated with better access to transplantation (HR 0.96 [95% CI 0.90–1.02]), although this relation differed by baseline body mass index and for recipients of living versus deceased donor organs. For example, weight loss was associated with improved access to living donor transplantation (HR 1.24 [95% CI 1.07–1.44]) in whites but not in blacks or Hispanics. In a cohort of obese patients waitlisted before dialysis, blacks were more likely to lose weight and less likely to gain weight compared with whites. Weight loss was only associated with improved access to living donor transplantation among whites. Further studies are needed to understand the reasons for the observed associations.

## Introduction

Obesity has increased in prevalence among adults with end-stage kidney disease (ESKD) at a rate that has exceeded that of the general population [1–3]. The high prevalence of obesity among patients with ESRD is concerning because many transplant centers in the US continue to have pre-specified body mass index (BMI) criteria for candidates to be deemed eligible for transplantation [4–7]. Although some guidelines no longer recommend use of BMI cutoffs for transplant eligibility determination [8,9], many transplant centers in the US have not yet adopted these recommendations. A number of studies have shown that excess weight affects access to living and deceased donor transplantation among those who are on the kidney transplant waitlist [4,10,11]. Although excess weight is associated with short-term complications after transplantation such as impaired wound healing or re-hospitalization, the relationship between having excess weight before transplantation and worse long-term allograft or patient survival after transplantation has been inconsistent and debated [12–19]. Hence, some guidelines continue to recommend achievement of healthier weight status prior to kidney transplantation [8].

Most studies that have examined the role of BMI as a barrier to transplantation have used one-time measurements of BMI [4,10]. Fewer studies have examined weight changes after waitlist registration or whether weight loss improves transplantation rates among traditionally disadvantaged groups who are known to have lower access to transplantation [20–22]. For example, black patients with diabetes are more obese than white patients at the time of dialysis initiation [1]. Thus, it is conceivable that disparities in transplantation may be related to racial and ethnic differences in the ability to meet BMI criteria for kidney transplantation.

Our objective was to ascertain whether there are racial or ethnic differences in time to receipt of a kidney transplant among obese candidates who were waitlisted prior to receiving renal replacement therapy. We hypothesized that non-Hispanic black (NHB) and Hispanic patients registered on the waitlist would be less likely to lose weight compared to non-Hispanic whites (NHWs). We also hypothesized that when weight loss did occur, it would improve transplantation rates compared to maintenance of stable weight or weight gain, especially among the NHB and Hispanic populations that have higher BMI on average than NHW patients.

## Methods

### Study population and data source

**Fig 1:**
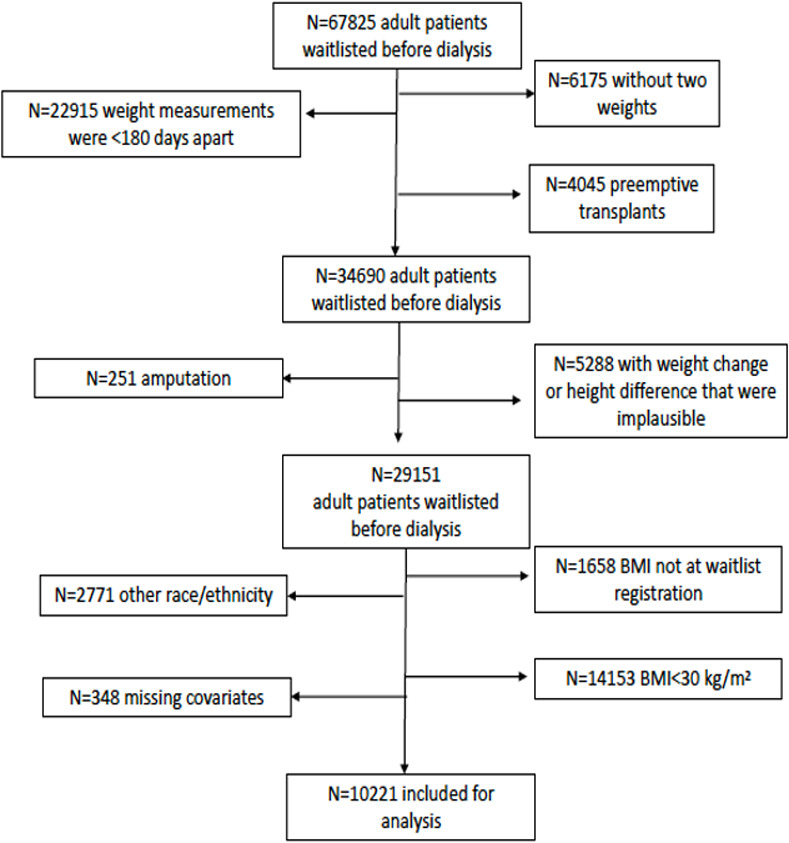
Consort diagram.

We included adults 18 years of age or older who were registered on the kidney transplant waitlist between 1995–2015 before receiving treatment for ESKD and who had a BMI ≥30 kg/m^2^ (and were therefore obese) at the time of waitlist registration. We only included patients who were identified as NHW, NHB, or Hispanic in the Patients file of the United States Renal Data System (USRDS), given the smaller number of patients of other racial or ethnic backgrounds. We required that the first weight measurement be taken at waitlist registration prior to dialysis initiation to ensure that the population included for study would be more homogenous (since those patients who were waitlisted after dialysis initiation may differ from those preemptively waitlisted). The second weight was typically the weight reported at the time of dialysis initiation. We excluded patients who did not have two weight and height measurements that were at least 180 days apart (Fig 1). We excluded patients who received a kidney transplant on the same day as their second weight measurement (i.e. preemptive transplants) because the only available second weight was typically obtained at the time of ESRD onset (and therefore at the time of preemptive transplantation), which precluded the inclusion of these patients. We also excluded patients with known amputations, and patients with implausible weight or height measurements (see below). Details surrounding our cohort derivation are shown in Fig 1.

The Committee on Human Research of the University of California San Francisco considers this work not to be human subjects research. Consent was not obtained as the data was analyzed anonymously.

### BMI ascertainment

Weight and height at waitlist registration were used to calculate BMI in kilograms per meter squared. Weight and height are reported to the USRDS (or United Network for Organ Sharing from which USRDS derives data for its WAITLIST and TRANSPLANT files) at the time of transplant waitlist registration and dialysis initiation. If height was missing, we allowed the inclusion of height measurements obtained after waitlist registration. We set height values >213 cm and weight values <30 kg as missing given that we thought these values were likely to be erroneous and implausible. If there was more than one height available in the USRDS and they were within 5 cm of each other, the average of the heights was taken. If there were more than two heights and the difference between heights was >5 cm, the outlier was excluded and the two heights closest to each other were averaged. We then compared the two available weights in our cohort. If there was more than a two fold difference between weights we investigated whether these were kilogram (kg) to pound conversion reporting errors by comparing these weights with other reported weight and heights from different dates for the same patient; when such discrepancies were detected, the weight was converted to the appropriate units (pounds to kg or vice versa). We classified the baseline BMI (defined as the first BMI at the time of waitlist registration) into the following categories: 30-<35 kg/m^2^, 35-<40 kg/m^2^, or ≥40 kg/m^2^ in our primary analyses.

Because height remains stable in adults, we considered BMI a reflection of weight status and henceforth refer to changes in weight and BMI interchangeably.

### Weight change category

We assessed weight change by computing the annualized weight change, taking the difference between the two BMI measurements divided by the time between these measurements to obtain the mean change in BMI per year. If more than two weights were available, we took the weight at waitlist registration and the chronologically most recent weight available for computation of the mean change in BMI per year. We categorized weight changes according to BMI (and not absolute weight) because the impact of an absolute change in weight would be expected to differ for smaller versus larger individuals, and because decisions about transplant eligibility generally hinge on BMI. Stable weight was considered to be a BMI change of -2 to +2 kg/m^2^/year, whereas weight gain and weight loss were considered to be BMI changes greater than 2 kg/m^2^/year in either direction. We used these definitions because a prior study on this issue demonstrated that among waitlisted patients whose main barrier was known to be obesity [19], the median weight loss achieved among those who ultimately received a transplant was 2 kg/m^2^.

### Outcome ascertainment

Our primary outcome was receipt of a transplanted kidney (overall and by living or deceased donor transplantation). We chose to focus on actual receipt of a transplanted kidney as our primary outcome in order to capture all potential barriers associated with excess weight between the time of waitlist registration and transplantation (e.g., such as concerns about size mismatch and bypassing of organ offers for obese patients after waitlist activation) [23]. Furthermore, prior studies have shown that even after waitlist activation, obese patients are still less likely to receive a transplanted kidney [4]. We determined transplant dates and donor source (living versus deceased) for first transplant using USRDS Patient and Transplant files, which contain data reported by transplant centers to the United Network for Organ Sharing. We abstracted death dates from the USRDS Patients file. We ascertained all outcomes (death and transplants) through June 30, 2016.

### Statistical analyses

To determine whether there were differences in the characteristics of patients by weight change category at the time of waitlist registration, we used Chi-square, Wilcoxon rank sum, or Kruskal-Wallis tests.

#### Race/Ethnicity and likelihood of weight change

We used multinomial logistic regression models to examine the association between race/ethnicity and weight change category (stable, loss, or gain). In these models, NHWs were considered the reference group for the primary predictor, and stable weight was considered the reference group for the outcome. We used unadjusted and adjusted models to compare the odds of weight loss or weight gain among NHWs, NHBs, and Hispanics. In our adjusted analyses, we included age at the second BMI measurement, sex, race category, diabetes, coronary artery disease, heart failure, cancer, tobacco, median neighborhood income, dialysis modality (hemodialysis versus peritoneal dialysis) and UNOS region by patient state of residence in our models (Model 1). We then adjusted additionally for baseline BMI (Model 2) to account for potential differences in the starting weight.

#### Association between BMI change and access to transplantation

We first assessed the global association between weight change category (stable, loss, or gain) and access to transplant (living or deceased donor as a composite outcome) using unadjusted and adjusted Fine-Gray models, adjusting for the same covariates as described in Model 1. We examined this association for the overall cohort and treated death as a competing risk for transplantation. In these models, time began at the date of the second BMI measurement (which typically was at the time of dialysis initiation) since we only included patients who were waitlisted before dialysis initiation. Follow-up was censored at the time of death, transplantation, or administratively as of June 30, 2016.

Next, we tested for interactions between 1) race/ethnicity and BMI change category and 2) baseline BMI category (i.e. 30–35 kg/m^2^, 35–40 kg/m^2^, and >40 kg/m^2^) and BMI change category. Because of the presence of an interaction between baseline BMI category and BMI change, we repeated our Fine-Gray models and stratified our analyses by baseline BMI category using adjusted models (Model 1).

In sensitivity analyses, and to further refine whether weight change was associated with better access to transplantation among those with borderline BMI (e.g., a patient with a BMI of 36 kg/m^2^ may become eligible for transplantation if their BMI drops to 35 kg/m^2^), we re-categorized BMI by smaller increments of 2.5 kg/m^2^ and repeated our Fine-Gray models, recognizing that the smaller number of patients in each of these refined BMI categories may lead to lesser power.

Next, to determine whether the association between weight change and transplantation differed by race/ethnicity (which was our *a priori* hypothesis), we also constructed a nine-category predictor, taking into account both race/ethnicity (NHW, NHB, or Hispanic) and BMI change (stable, weight gain, or weight loss). We considered NHWs with stable weight as the reference group for this nine-category predictor and examined its association with living or deceased donor transplantation using separate Fine-Gray models, adjusting for the same covariates as above (Model 1 and Model 2). For models where the outcome was living donor transplantation, death and deceased donor transplantation were treated as competing risks. For models where the outcome was deceased donor transplantation, death and living donor transplantation were treated as competing risks. We considered Model 2 our primary models in all analyses, except when we found interactions between the primary predictor (BMI change) and baseline BMI.

All analyses were performed using SAS 9.4. The Committee on Human Research of the University of California San Francisco considers this work not to be human subjects research. USRDS data are available upon request to the public at www.usrds.org. Consent was not obtained as the data were retrospectively analyzed in de-identified form. We adhered to reporting guidelines recommended by RECORD for observational studies.

## Results

### Study participants

We included 10 221 obese patients who were waitlisted for kidney transplantation prior to starting dialysis in our analyses. Over half of this cohort was NHW, 27% NHB, and 13% Hispanic. Approximately 46% of patients underwent transplantation (12% from a living donor), and 14% died during 1.6 years of median follow-up [interquartile range (IQR) 0.65–2.99 years].

### Weight changes by race/ethnicity

We first examined characteristics of our study population by weight change category. For those who met our inclusion criteria, 29% experienced weight loss and 7% weight gain (Table 1). Among patients who lost weight, a larger proportion were NHB. Among those who gained weight, a larger proportion were NHW. Patients who gained weight tended to be younger at the time of their second weight measurement compared to those who lost or maintained weight. Men were more likely to maintain stable weight compared to women. The median and interquartile range (IQR) in time between the first and last BMI measurement used to compute weight change was 443 [IQR 287–743] days.

**Table 1:** Baseline cohort characteristics by weight change category.

Column % or Mean ± SD unless otherwise specified	Overall (N = 10 221)	Lost weight (N = 2 927)	Stable Weight (N = 6 602)	Gained weight (N = 692)
Age at second weight (years)** [IQR]	57 [47,64]	57 [48, 64]	57 [48, 65]	53 [45, 61]
Male**	59	57	60	53
Race/ethnicity**				
NHW	60	56	61	63
NHB	27	31	26	23
Hispanic	13	13	13	13
Initial mean BMI** (kg/m^2^) ± SD	35 ± 4	35 ± 4	34 ± 4	34 ± 4
Follow-up mean BMI** (kg/m^2^) ± SD	33 ± 4	31 ± 4	34 ± 4	38 ± 5
Inactive at waitlist	35	35	35	33
Median income**	53 609	52 302	53 220	51 433
[IQR]	[42 027–70 973]	[40 670–69 077]	[41 936–69 859]	[41 177–68 895]
Diabetes**	40	41	40	42
CAD	9	9	9	6
Heart failure**	11	10	10	11
Cancer	4	3	4	4
Tobacco use	2	3	2	3
In-center Hemodialysis**	74	81	72	69

**Statistically significantly different (p<0.05) compared across weight change categories.

CAD = coronary artery disease; SD = standard deviation; BMI = body mass index.

Compared to NHWs, NHBs had a 24% higher odds of losing weight (95% CI 1.12–1.38) and Hispanics had a 8% higher odds of losing weight (95% CI 0.93–1.25) that did not achieve statistical significance in our primary adjusted analysis (Model 2, Table 2).

**Table 2:** Odds of weight gain or weight loss by race/ethnicity in multinomial logistic models.

	Gained weight N = 692	Lost weight N = 2 927
**Unadjusted**		
NHB	0.87 (0.72–1.05)	1.31 (1.19–1.45)
NHW	Ref	Ref
Hispanic	1.02 (0.81–1.30)	1.10 (0.96–1.26)
**Model 1**		
NHB	0.77 (0.63–0.94)	1.26 (1.13–1.40)
NHW	Ref	Ref
Hispanic	0.95 (0.73–1.23)	1.06 (0.91–1.23)
**Model 2**		
NHB	0.78 (0.64–0.95)	1.24 (1.12–1.38)
NHW	Ref	Ref
Hispanic	0.95 (0.73–1.23)	1.08 (0.93–1.25)

**Model 1.** Adjusted for age at second weight measurement, sex, race category, diabetes, coronary artery disease, congestive heart failure, cancer, smoking, median neighborhood income, UNOS region, dialysis modality.

**Model 2.** Model 1 + baseline BMI.

Reference group = Stable weight (N = 6602).

Compared with NHWs, NHBs were statistically significantly less likely to gain weight in our primary models (Model 2). Hispanics had a lower tendency to gain weight that did not achieve statistical significance.

### Association between weight changes and transplantation by race/ethnicity

A total of 1 240 living donor and 3 482 deceased donor transplants occurred during follow-up. Death prior to transplantation was more common among those who lost weight (15%) or gained weight (15%) compared with those who maintained stable weight (13%).

Overall, patients who lost weight did not statistically significantly differ from those who maintained their weight in their risk of kidney transplantation in our primary analysis (Table 3). Patients who gained weight were less likely to receive a transplanted kidney compared with those who maintained stable weight.

**Table 3:** Fine-Gray models of the association between weight change category and risk of transplant (deceased or living donor) by baseline BMI, treating death as a competing risk.

Model 1* Sub-hazard ratio (95% CI)	Overall N = 10 221	30-<35 kg/m^2^ N = 6 380	35-<40 kg/m^2^ N = 2991	≥40 kg/m^2^ N = 850
Weight loss	0.96 (0.90–1.02)	0.92 (0.85–1.003)	1.08 (0.96–1.22)	1.10 (0.87–1.40)
Stable weight	Reference	Reference	Reference	Reference
Weight gain	0.88 (0.79–0.99)	0.94 (0.82–1.07)	0.81 (0.64–1.02)	0.61 (0.36–1.01)

*Adjusted for age at second weight measurement, sex, race category, diabetes, coronary artery disease, congestive heart failure, cancer, smoking, median neighborhood income, UNOS region, and dialysis modality; p <0.001 for interaction between baseline BMI and BMI change.

CI = confidence interval.

We tested for and found a statistically significant interaction between BMI category at baseline and BMI change category (p<0.001) but not between race/ethnicity and BMI change category (p = 0.22). Thus, we stratified our overall analysis by baseline BMI categories (Table 3). Among waitlisted individuals with weight loss, the risk of transplant tended to higher with increasing baseline BMI even though the point estimate did not achieve statistical significance. Among waitlisted individuals with weight gain, risk of transplantation tended to decrease as baseline BMI increased (Table 3).

In sensitivity analysis, when we refined our BMI categories by smaller increments, similar trends persisted (S1 Table).

### Weight changes by race/ethnicity and living donor transplantation

**Fig 2:**
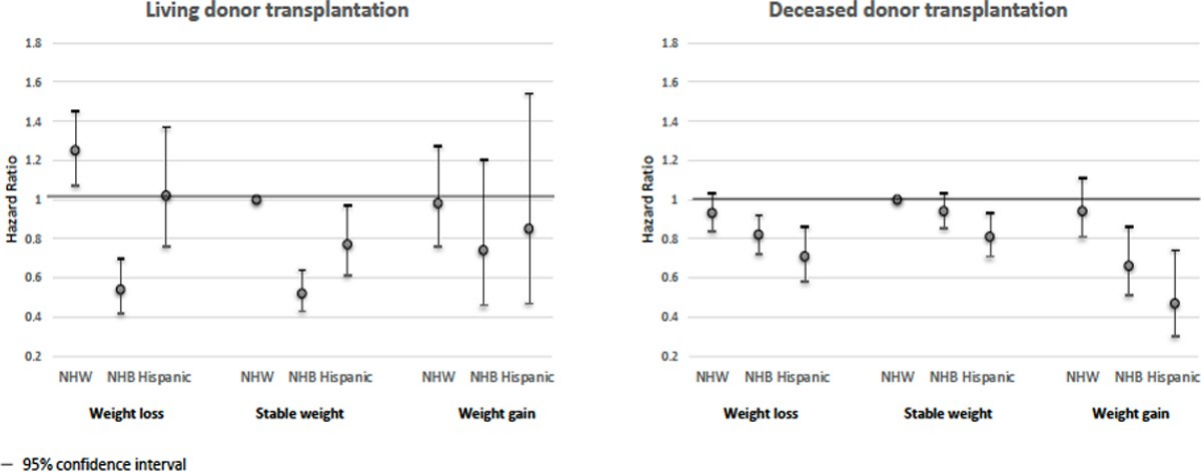
Access to living or deceased donor transplantation using adjusted Fine-Gray models.

When we examined the association between our nine-category predictor and access to living donor transplantation, we found that weight loss was associated with higher hazard of living donor transplantation in NHWs (HR1.24 [95% CI 1.07–1.44]), similar risk in Hispanics (HR 1.02 [95% CI 0.76–1.36]), and lower hazard in NHBs (HR 0.54 [95% CI 0.41–0.70]) compared with the reference group (NHWs with stable weight, Model 2, Fig 2 and S2 Table).

Both NHBs and Hispanics who maintained stable weight had lower hazard of living donor transplantation compared with NHWs who maintained stable weight (S2 Table and Fig 2), but this finding was especially pronounced when comparing NHBs with NHWs (Model 2, HR 0.52 [95% CI 0.43–0.63]).

Among NHWs, NHBs, and Hispanic patients who gained weight, hazard of living donor kidney transplantation was not statistically significantly different compared with the reference group (NHWs with stable weight, S2 Table and Fig 2).

### Weight changes by race/ethnicity and deceased donor transplantation

When we repeated our Fine-Gray models using our nine-category predictor to examine the risk of deceased donor transplantation, we found that NHWs with stable weight had the highest hazard of deceased donor transplantation (Fig 2 and S3 Table).

Across all racial/ethnic groups, weight loss was associated with lower hazard of deceased donor transplantation compared to NHWs with stable weight (S3 Table and Fig 2). Weight gain was also associated with lower hazard of deceased donor transplantation in both NHBs and Hispanics, but in NHWs.

Among those with stable weight, the disparity in risk of deceased donor transplantation was more pronounced when comparing Hispanics with NHWs (HR 0.81 [95% CI 0.71–0.93]) and less pronounced (but not statistically significantly different) when comparing NHBs with NHWs (HR 0.93 [95% CI 0.85–1.03], Fig 2).

## Discussion

The optimal approach to weight management in obese kidney transplant candidates has been debated for many decades [5–9,15,16,24–26]. Multiple observational studies have demonstrated that obesity is associated with lower risk of mortality in dialysis patients [27–32]. Yet at the same time, transplantation is more limited among patients with excess weight, given that many transplant centers continue to maintain BMI criteria that recipients must meet to remain eligible for transplantation [6,7,33,34]. This often poses a dilemma for providers caring for dialysis patients who are potentially eligible for kidney transplantation regarding appropriate weight counseling [35]. Our goal was to examine differences in receipt of transplantation by race/ethnicity based on weight changes observed between the time of waitlist registration and dialysis initiation in an obese cohort. We chose to focus on this cohort because we believed these patients would be healthier than the rest of the ESRD population given their preemptive waitlist registration, and therefore our observed weight changes may be less attributable to declines in health status. Indeed, our cohort was relatively young, had a lower prevalence of comorbid conditions than the general dialysis population, and had high transplantation rates during follow-up.

To our surprise, we found that NHBs who were obese at baseline were more likely to lose weight and less likely to gain weight after waitlist registration compared with NHWs. Among those who lost weight, there was a trend toward better access to transplantation at higher BMI. Among those who gained weight, there was a trend toward lower access to transplantation at higher BMI. Whereas weight loss was associated with greater risk of living donor transplantation in NHWs (vs. maintenance of stable weight), this was not true for NHBs or Hispanics. NHBs and Hispanics also had consistently lower access to deceased donor transplantation compared to NHWs, regardless of weight changes that occurred.

We believe our study to be one of the first to examine the relationship between weight changes and kidney transplantation in an obese population within a context that considers the known racial and ethnic disparities in access to transplantation [36]. The majority of the available literature surrounding this issue has used one-time measures of BMI for the evaluation of the association between weight status and access to transplantation or allograft outcomes [10,17,37]. A recent study of French patients noted that every 10% weight loss among dialysis patients was associated with improved access to transplantation, suggesting that body size remains a barrier to transplantation even at transplant programs outside of the US [11]. Our findings are consistent with the observation in this French study in that weight loss did tend to increase transplantation rates, especially from a living donor among NHWs according to our results. However, weight loss was not associated with higher risk of living donor transplantation among other racial/ethnic groups, and weight loss did not improve risk of deceased donor transplantation for any racial/ethnic groups, including NHWs.

A prior study showed that patients treated with peritoneal dialysis were less likely to gain weight compared to patients treated with hemodialysis [38]. Our data suggest that weight gain may serve as a significant barrier to transplantation across racial and ethnic groups, although our cohort consists mostly of patients treated with hemodialysis after ESRD onset. Importantly, a few recent studies have suggested that bariatric surgery may be a safe option in the ESKD population that can help individuals achieve intentional weight loss. Such weight loss was associated with improved access to kidney transplantation [33,34], although not all obese patients treated with dialysis may be candidates for such a procedure.

Our findings did differ by donor source. Weight loss was associated with higher risk of living donor transplantation among NHWs but not other racial or ethnic groups. Although we do not have data on whether this weight loss was volitional, given the overall health of our cohort and the potential greater motivation to lose weight in a pre-emptively waitlisted population, we believe that it is likely that the observed weight loss was not necessarily due to greater severity of illness. However, the reasons why weight loss in other racial/ethnic groups did not also improve risk of living donor transplantation are unclear. It is likely that the barriers to living donor transplantation for NHB and Hispanic patients may lie in the lack of availability of living donors, as opposed to inability of recipients to achieve BMI criteria for transplantation [22,39–41].

Obese patients on the kidney transplant waitlist are frequently counseled to lose weight due to concerns surrounding post-transplant complications, including higher rates of delayed graft function, wound complications, acute rejection, and higher rates of graft loss [37,42,43]. However, much of the literature reporting on the risk of post-operative complications among those who were obese at the time of transplantation has focused on one-time weight measurements rather than assessments of weight changes. Of the studies that have examined associations between pre-transplant weight loss and graft failure or mortality risk, results have been mixed, with some studies indicating that pre-transplant weight loss was not associated with differences in allograft or patient survival [44] and others showing higher risk of graft loss and all-cause hospitalizations [45]. Given that weight loss is associated with higher risk of complications after deceased donor transplantation [45] *and* higher BMI is associated with better survival on dialysis [27–32] *and* weight loss does not associate with higher rates of transplantation (except in NHWs with living donors), it is unclear whether BMI should remain a criterion for recipient eligibility for transplantation (and therefore encouragement of weight loss to achieve a lower BMI).

The strengths of our study include the large size of the national cohort, the contemporary nature of the data, and the inclusion of a racially and ethnically diverse group of patients. Limitations include potential errors in data used for BMI determination and missing data from the Kidney Transplant Registration or CMS-2728 forms that may have led to potential misclassification of BMI category or excluded some patients from our study. We are limited in our ability to account for the potential presence of fluid overload in our BMI determination, although we believe that in clinical practice, edema is also not factored into BMI criteria for eligibility for kidney transplantation, and the presence of edema would only bias our results towards the null (as those who lost weight likely lost more true body weight than we estimated if they also developed worsening fluid overload with advancing kidney disease). We are also unable to distinguish between changes in muscle mass versus adipose tissue. Another limitation is that patients may experience weight changes prior to waitlist registration and not be evaluated for potential kidney transplantation if they are not within the BMI criteria needed for transplantation. As such, our findings may not generalize to the dialysis population not registered on the transplant waitlist prior to the initiation of dialysis. We do not have data on organs that were offered but not transplanted in our database. Obese recipients may have other reasons for lack of access to transplantation, including fewer potential eligible donors who are healthy enough to serve as living donors, but our study does not capture data surrounding the number of ineligible donors. In addition, we cannot ascertain whether weight loss might have been related to illness. Residual confounding may be present.

In conclusion, weight loss was more common among obese NHB and Hispanic patients registered on the kidney transplant waitlist prior to initiating dialysis, but this weight loss was not associated with access to transplantation in these traditionally disadvantaged groups. The disparities in access to transplantation among NHB and Hispanic patients do not appear to be due to differential ability to achieve weight loss (regardless of whether this weight loss is volitional). Given that weight loss may also be associated with worse patient and allograft survival post-transplantation and does not consistently associate with improved access to transplantation, transplant centers may consider liberalization of body size criteria when considering transplant candidacy. However, there may be other benefits to weight loss (including better cardiovascular health) that should be considered. A more concerted effort is needed to understand additional barriers to access to transplantation by race and ethnicity.

## Supporting information

S1 Checklist The RECORD statement–checklist of items, extended from the STROBE statement, that should be reported in observational studies using routinely collected health data. (DOCX)

S1 Table Fine-Gray models for the association between weight change category and transplantation by BMI increments of 2.5 kg/m^2^. (DOCX)

S2 Table Fine-Gray models of the association between weight change category and risk of living donor transplant, treating deceased donor transplantation and deaths as competing risks. (DOCX)

S3 Table Fine-Gray models of the association between weight change category and risk of deceased donor transplant, treating living donor transplantation and deaths as competing risks. (DOCX)
