# Supplementary material for: Weighing the waitlist: Weight changes and access to kidney transplantation among obese candidates
Source: PLoS One. 2020 Nov 30;15(11):e0242784. doi: 10.1371/journal.pone.0242784 (PMC7703917; doi:10.1371/journal.pone.0242784)
Supplement: S1 Table — (DOCX) [file pone.0242784.s002.docx]

**S1 Table. Fine-Gray models for the association between weight change category and transplantation by BMI increments of 2.5 kg/m^2^**

| **Model 1*** | **30-<32.5 kg/m^2^** | **32.5-<35 kg/m^2^** | **35-<37.5 kg/m^2^** | **37.5-<40 kg/m^2^** | **40-<42.5 kg/m^2^** | **≥42.5 kg/m^2^** |
| --- | --- | --- | --- | --- | --- | --- |
| Weight loss  Stable weight  Weight gain | 0.87 (0.77-0.97)  Reference  1.04 (0.88-1.24) | 1.01 (0.89-1.14)  Reference  0.84 (0.68-1.03) | 1.09 (0.94-1.27)  Reference  0.80 (0.61-1.06) | 1.05 (0.85-1.29)  Reference  0.73 (0.48-1.12) | 1.26 (0.91-1.73)  Reference  0.71 (0.38-1.32) | 1.10 (0.74-1.62)  Reference  0.32 (0.10-1.01) |

***Model 1.** Adjusted for age at second weight measurement, sex, race category, diabetes, coronary artery disease, congestive heart failure, cancer, smoking, median neighborhood income, OPTN region, dialysis modality
