# Supplementary material for: Weighing the waitlist: Weight changes and access to kidney transplantation among obese candidates
Source: PLoS One. 2020 Nov 30;15(11):e0242784. doi: 10.1371/journal.pone.0242784 (PMC7703917; doi:10.1371/journal.pone.0242784)
Supplement: S2 Table — (DOCX) [file pone.0242784.s003.docx]

**S2 Table. Fine-Gray models of the association between weight change category and risk of living donor transplant, treating deceased donor transplantation and deaths as competing risks.**

| N=10 222  Sub-hazard ratio (95% CI) | **Weight loss** | **Stable weight category** | **Weight gain** |
| --- | --- | --- | --- |
| **Unadjusted** | | | |
| NHW | 1.21 (1.04-1.40) | Reference | 1.06 (0.82-1.37) |
| NHB | 0.52 (0.40-0.67) | 0.52 (0.42-0.63) | 0.73 (0.45-1.19) |
| Hispanic | 1.01 (0.76-1.34) | 0.81 (0.65-1.01) | 0.96 (0.54-1.70) |
| **Model 1** | | | |
| NHW | 1.21 (1.04-1.40) | Reference | 0.98 (0.76-1.27) |
| NHB | 0.52 (0.40-0.67) | 0.51 (0.42-0.63) | 0.75 (0.46-1.21) |
| Hispanic | 1.01 (0.76-1.35) | 0.78 (0.62-0.98) | 0.88 (0.49-1.59) |
| **Model 2** | | | |
| NHW | 1.24 (1.07-1.44) | Reference | 0.98 (0.76-1.27) |
| NHB | 0.54 (0.41-0.70) | 0.52 (0.43-0.63) | 0.75 (0.46-1.21) |
| Hispanic | 1.02 (0.76-1.36) | 0.77 (0.61-0.97) | 0.86 (0.48-1.56) |
