# Supplementary material for: Weighing the waitlist: Weight changes and access to kidney transplantation among obese candidates
Source: PLoS One. 2020 Nov 30;15(11):e0242784. doi: 10.1371/journal.pone.0242784 (PMC7703917; doi:10.1371/journal.pone.0242784)
Supplement: S3 Table — (DOCX) [file pone.0242784.s004.docx]

**S3 Table. Fine-Gray models of the association between weight change category and risk of deceased donor transplant, treating living donor transplantation and deaths as competing risks.**

| N=10 221  Sub-hazard ratio (95% CI) | **Weight loss** | **Stable weight category** | **Weight gain** |
| --- | --- | --- | --- |
| **Unadjusted** | | | |
| NHW | 0.92 (0.83-1.01) | Reference | 0.98 (0.83-1.15) |
| NHB | 0.82 (0.73-0.92) | 0.96 (0.88-1.06) | 0.69 (0.53-0.90) |
| Hispanic | 0.63 (0.52-0.76) | 0.74 (0.65-0.84) | 0.45 (0.28-0.70) |
| **Model 1** | | | |
| NHW | 0.90 (0.82-1.00) | Reference | 0.94 (0.80-1.10) |
| NHB | 0.79 (0.70-0.89) | 0.93 (0.84-1.02) | 0.67 (0.51-0.87) |
| Hispanic | 0.69 (0.57-0.84) | 0.82 (0.71-0.94) | 0.48 (0.31-0.74) |
| **Model 2** | | | |
| NHW | 0.92 (0.84-1.02) | Reference | 0.95 (0.81-1.11) |
| NHB | 0.81 (0.72-0.92) | 0.93 (0.85-1.03) | 0.66 (0.51-0.86) |
| Hispanic | 0.70 (0.57-0.85) | 0.81 (0.71-0.93) | 0.47 (0.30-0.73) |

**Model 1.** Adjusted for age at second weight measurement, sex, race category, diabetes, coronary artery disease, congestive heart failure, cancer, smoking, median neighborhood income, OPTN region, dialysis modality

**Model 2.** Model 1 + Baseline BMI.

CI = confidence interval
